# Genome Sequence of the Oleaginous Green Alga, *Chlorella vulgaris* UTEX 395

**DOI:** 10.3389/fbioe.2018.00037

**Published:** 2018-04-05

**Authors:** Michael T. Guarnieri, Jennifer Levering, Calvin A. Henard, Jeffrey L. Boore, Michael J. Betenbaugh, Karsten Zengler, Eric P. Knoshaug

**Affiliations:** ^1^National Bioenergy Center, National Renewable Energy Laboratory, Golden, CO, United States; ^2^Division of Host-Microbe Systems and Therapeutics, Department of Pediatrics, University of California, San Diego, La Jolla, CA, United States; ^3^Genome Project Solutions, Inc., Hercules, CA, United States; ^4^Department of Chemical and Biomolecular Engineering, Johns Hopkins University, Baltimore, MD, United States

**Keywords:** microalgae, biofuels, lipids, genome, *Chlorella vulgaris*

## Introduction

Microalgae have garnered extensive interest as renewable fuel feedstocks due to their high production potential relative to terrestrial crops, and unique cultivation capacity on non-arable lands (Wijffels and Barbosa, 2010; Davis et al., 2011). The oleaginous chlorophyte *Chlorella vulgaris* represents a promising model microalgal system and production host, due to its ability to synthesize and accumulate large quantities of fuel intermediates in the form of storage lipids (Guarnieri et al., 2011, 2012; Gerken et al., 2013; Griffiths et al., 2014; Zuñiga et al., 2016). Recent omic analyses have identified transcriptional, post-transcriptional and -translational mechanisms governing lipid accumulation in this alga (Guarnieri et al., 2011, 2013), including active protein nitrosylation (Henard et al., 2017). Here we report the draft nuclear genome and annotation of *C. vulgaris* UTEX 395.

## Materials and methods

### Cultivation and genomic DNA isolation

For genomic DNA isolation *C. vulgaris* UTEX 395 was grown photoautotrophically to exponential phase in Bold's Basal Media, under constant illumination (200 μE m^−2^ s^−1^ white fluorescent light), and supplemented with 2% CO_2_/air, as described previously (Guarnieri et al., 2011, 2013). Genomic DNA was extracted following the protocol adapted from Varela-Alvarez et al. (2006).

### Genome sequencing and assembly

Sequencing was performed using Illumina HiSeq 2000 technology with 108 cycles. 171,758,456 paired-end (SIPES) reads were trimmed to an error rate of <1:100, then trimmed until no ambiguous nucleotides remain; reads shorter than 20 nucleotides were discarded, retaining 168,611,711 reads, of which 165,874,962 remained as pairs. Resultant reads were assembled using a DeBruijn method; 113 scaffolds were generated at ≥1,000x depth of coverage, 24 of which were longer than 100 kb and 566 of which were 20–100 kb, ultimately generating a total assembly size of 37.34 Mb, with a 61.5% GC content. This represents the smallest nuclear genome size and lowest GC content reported to date for a sequenced *Chlorella* species (Supplemental Table 1).

### Genome annotation

Transcript prediction was conducted using Maker (Cantarel et al., 2008; Campbell et al., 2014). Transcripts were six-frame translated into protein sequences and functionally annotated with EC, GO and InterProScan identifiers using two approaches. First, a bidirectional BLASTp against SwissProt sequences was carried out and paralogs were identified using BLASTclust. Secondly, InterProScan and PRIAM analyses with gene and genome-specific profiles were conducted.

To facilitate refined annotation and comprehensive pathway mapping of *C. vulgaris*, a draft nuclear genome sequence was generated and integrated with previously acquired *de novo* transcriptomic datasets (Guarnieri et al., 2011). 7,100 transcripts were predicted from the *C. vulgaris* genome, resulting in 6,056 annotated gene models. Genomic queries identified complete gene sets encoding fatty acid and triacylglyceride biosynthetic pathways. The nitrogen assimilation inventory includes genes for nitrate/nitrite transporters and reductases. The genome also encodes meiosis-associated DMC1 and Rad51 DNA recombinase homologs (Fanning et al., 2006; Broderick et al., 2010), offering a possibility that sexual mating may occur in this microalga. Genes for the synthesis of the global stress response alarmone, guanine tetraphosphate (ppGpp) (Takahashi et al., 2004; Tozawa and Nomura, 2011), were also identified. Combined, these genetic pathways will enable potential markerless strain-engineering strategies targeting lipid accumulation in the absence of stress induction, ultimately facilitating the development of robust, deployment-viable microalgae for cost-competitive biofuel production.

## Direct link to deposited data and information to users

This whole-genome project has been deposited at DDBJ/EMBL/GenBank under the accession LDKB00000000. The version described in this paper is version LDKB01000000. Additional details can be found at http://www.nrel.gov/biomass/proj_microalgal_biofuels.html and http://chlorella.genomeprojectsolutions-databases.com.

## Author contributions

The work was designed by EK, MB, KZ, and MG. MG directed wet lab analyses. JB directed genome assembly. JL and CH directed genome annotation and pathway mapping.

### Conflict of interest statement

The authors declare that the research was conducted in the absence of any commercial or financial relationships that could be construed as a potential conflict of interest.
