# Supplementary material for: Genome Sequence of the Oleaginous Green Alga, Chlorella vulgaris UTEX 395
Source: Front Bioeng Biotechnol. 2018 Apr 5;6:37. doi: 10.3389/fbioe.2018.00037 (PMC5895722; doi:10.3389/fbioe.2018.00037)
Supplement: Supplementary file 1 [file Table1.DOCX]

**Supplemental Table 1. Genome summary for *Chlorella* species sequenced to date.**

| Species | Genome Size (Mb) | GC% |
| --- | --- | --- |
| *C. pyrenoidosa* FACHB-9 | 56.99 | 64 |
| *C. sorokiniana* 1602 | 59.57 | 64 |
| *C. sorokiniana* 1228 | 61.39 | 63.5 |
| *C. sorokiniana* UTEX 1230 | 58.53 | 63.6 |
| *C. variabilis* NC64A | 46.16 | 65.5 |
| *C. vulgaris* UTEX 395 | 37.34 | 61.5 |

Genome sizes were obtained from jgi.doe.gov and greenhouse.lanl.gov*.*
